# Videoconferences between remote-sitting specialist, patient and practice staff concerning low prevalent diseases and complex pathways in general practice clinics: a feasibility study

**DOI:** 10.1080/02813432.2026.2666626

**Published:** 2026-05-05

**Authors:** Mette Assenholm Kristensen, Hanne Irene Jensen, Christian Backer Mogensen, Jens Søndergaard, Jens Kjølseth Møller

**Affiliations:** a Department of Clinical Microbiology, Lillebaelt Hospital, University Hospital of Southern Denmark, Vejle, Denmark; b Department of Regional Health Research, University of Southern Denmark, Odense, Denmark; c Department of Anesthesiology and Intensive Care, Lillebaelt Hospital, University Hospital of Southern Denmark, Vejle and Kolding, Denmark; d Department of Emergency Medicine, Hospital Sønderjylland, University Hospital of Southern Denmark, Aabenraa, Denmark; e Research Unit of General Practice, Department of Public Health, University of Southern Denmark, Odense, Denmark

**Keywords:** Videoconference, general practice, hospital specialist, qualitative, feasibility

## Abstract

**Objectives:**

To develop a videoconference between a remote-sitting specialist, a patient and a practice staff (phase 1) and explore its feasibility in a small-scale real-world setting (phase 2) as preparation for a randomised controlled trial.

**Design:**

A feasibility study using participatory design with methicillin-resistant *Staphylococcus aureus* (MRSA) pathways as a case. The study was carried out between October 2020 and December 2022. In phase 1, we held a workshop with seven end-users. In phase 2, we carried out eight videoconferences and interviewed eight patients and seven practice staff who participated in the videoconferences. The analysis was performed according to Kvale and Brinkmann’s three interpretative contexts.

**Participants:**

Phase 1: One patient, general practitioners and healthcare providers with expertise in infection control and infectious diseases. Phase 2: Practice staff and Danish-speaking MRSA carriers planned to receive treatment and follow-up tests to confirm successful treatment.

**Results:**

Phase 1: The written description of the videoconference included tasks before, during and after the videoconference. It aimed to be patient-centered and to plan the patient pathway. Phase 2: During the analysis, three themes emerged: (1) ways to collaborate in standard care and videoconferences, (2) shared knowledge and planning of the pathway and (3) barriers when using videoconferences.

**Conclusion:**

The videoconferences are feasible and were perceived as acceptable, relevant and useful if they were structured, patient-centred and facilitated pathway planning. However, some patients found videoconferences less information-rich than face-to-face meetings, while some practice staff considered them time-consuming.

## Introduction

Staff in general practice clinics may find it challenging to develop high-quality work routines for low-prevalence diseases or complex care pathways rarely encountered by general practitioners (GPs). Infection or colonisation with methicillin-resistant *Staphylococcus aureus* (MRSA) exemplifies such challenges [1,2], in which a disease or condition affects a small fraction of patients and involves complicated guidelines to follow. MRSA care pathways may often require several treatments for both the patient and household members, including follow-up to monitor treatment effectiveness. Moreover, there are numerous guidelines for specific patient groups, which makes implementation in general practice challenging. A cohort study carried out in the Region of Southern Denmark found that only 36% of patients were successfully treated (MRSA-free) when managed in general practice. Furthermore, adherence to tests to confirm successful treatment was low [3]. Stigmatisation and the rates of cancellation of treatment and examinations in hospitals are relatively high for patients colonised with MRSA [4,5].

Research conducted by Dickmann et al. revealed that despite the accessibility of information regarding MRSA guidelines across various online platforms in Germany, both healthcare professionals and patients exhibited a preference for engaging in interactive discussions with specialists [6]. Hospital specialists’ e-mail consultation services are widely used to streamline care and improve access to specialists, typically within 48 h [7,8]. A downside of e-mail consultation is that it does not offer real-time problem-solving, does not include the patient, and is often inefficient for more complex care programs [9]. Specialist expertise is a limited resource. Videoconferences with various hospital specialists may be one effective approach to meet the need for interactive dialogue with patients and GPs. Very little research has been carried out within this field, but in a study, with patients with cancer, the GP and a hospital oncologist participated in a videoconference together with the patient. The patients reported high satisfaction, and the GPs and oncologists were overall positive, though to a lesser extent than the patients [10]. These cross-sector videoconferences between hospital specialists, practice staff and patients are still uncommon [11]. Our study aimed to develop a videoconference between a remote-sitting specialist, a patient and a practice staff (phase 1) and explore whether the videoconference was acceptable, relevant and useful for end-users, in a small-scale real-world setting (phase 2) as preparation for a randomised controlled trial.

## Material and methods

### Study design

**Figure 1.**
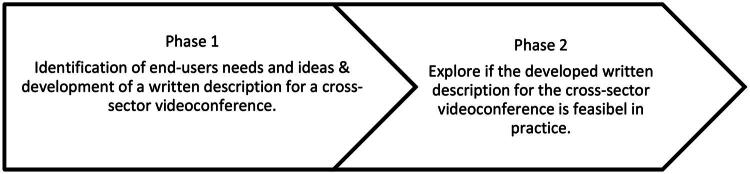
The phases of the study.

The study was carried out from October 2020 to December 2022 as a feasibility study using participatory design methodology. The method attempted to create a platform for active end-user participation in the development of new designs, including information technology [12]. Notably, both the videoconferencing and the setup with a remote-sitting infection preventionist (IP, first author) were predetermined by the research team. All authors contributed to the planning of the study, the final step of the analysis and the preparation of the manuscript. The first author was trained in qualitative research by Patient Data Explorative Network (OPEN). OPEN is a consultancy service for researchers, located in the Region of Southern Denmark. In the present study, we followed the participatory design methodology developed by Clemensen and colleagues as follows: needs assessment, idea generation, testing and retesting, and evaluation [13]. However, we only carried out needs assessment, idea generation and testing (not retesting). To develop the content and workflow for the cross-sector videoconference, we held a workshop, led by the first author, to identify needs and generate ideas (Phase 1). Afterwards, the developed content and workflow for the videoconference with general practice were carried out and evaluated by interviewing patients and practice staff (Phase 2). For details, see Figure 1.

The study was guided by the Standard for Reporting Qualitative Research (SRQR) guideline [14] and the CONSORT statement with an extension to randomised pilot and feasibility trials [15]. The study is part of a PhD Thesis [16].

### Setting and participants

The study was carried out in the Danish healthcare system in the Region of Southern Denmark. The Danish healthcare system is tax-funded, and all residents in Denmark are entitled to free or subsidised healthcare services, including general practice. In the Region of Southern Denmark, the staff at the general practice clinics manage the MRSA pathway and the Department of Clinical Microbiology assists by advising by phone. Advice is provided by either a clinical microbiologist or an IP. IPs in Denmark are generally nurses who have spent at least 2 years as registered healthcare providers and have had training and a certification exam covering microbiology, epidemiology, infection control and hospital hygiene. In the standard regional care model, practice staff (nurses or GPs) inform patients with MRSA, prescribe the decolonisation treatment of MRSA carriers and perform tests to confirm successful treatment.

The participating units were Lillebaelt Hospital, the Hospital of Southern Jutland and general practices in the catchment areas of these two hospitals in the Region of Southern Denmark. General practice clinics participated voluntarily in the study and were individually remunerated by the regional health department.

#### Phase 1

The author group identified healthcare professionals and a patient for the workshop who all accepted to participate. The purpose of recruitment was to provide a broad perspective on handling complex and rarely encountered medical conditions, in this case, MRSA colonisation. We applied the recommendations on suitable participants for participatory design processes, e.g. persons with an eye for detail and seeing the big picture [12]. The first author invited participants *via* e-mail.

#### Phase 2

We identified patients using a regional MRSA database comprising laboratory data on MRSA samples. Only individuals with a presumed complex pathway (expected decolonisation treatment and several tests to confirm successful treatment) could participate. Furthermore, participants had to be mentally capable of cooperating, understanding, speaking Danish and participating in the videoconference at the general practice clinic. An independent IP contacted the practice staff of eligible patients by phone. If the practice staff agreed to participate, they contacted the patients and provided them with oral information. The IP then provided oral project information to practice staff and written information to both patients and practice staff.

#### Equipment for the videoconference

We used the Cisco Webex meeting system for the videoconference. Practice staff used the system through a browser, which required no specific software or licences. The IP (first author) used a screen from Cisco (TRANBJERG DX80). The Cisco screen was connected to a computer by a DP-HDMI cable, enabling screen-sharing options. A unique link to the web-based videoconference was sent *via* email to the general practice clinics. To connect, the practice staff entered a unique meeting code. The system provided a secure connection with no third-party data processing. In most cases, the practice staff used a static computer combined with a microphone and speaker, along with a webcam attached to the top of their computer screens. Tablets, smartphones, or laptops could also be used.

### Data collection and data analyses

#### Authors’ motivations and preconceptions

The co-authors’ motivations and preconceptions include specific expertise in the investigation and detection of MRSA (JKM), MRSA decolonisation (JKM, JS), experience with qualitative research methods (HIJ), particular knowledge of workflows in general practice (JS) and participation in epidemiological studies (all co-authors). The first author has experience in providing advice to general practice, including MRSA decolonisation, infection prevention measures and initiatives to minimise stigmatisation.

#### Key concepts

In our study, we defined ‘acceptable’ as the extent to which end-users felt comfortable using the videoconference format and were satisfied with its content and delivery; ‘relevant’ as the extent to which the content addressed end-users’ perceived needs and clinical questions; and ‘useful’ as the extent to which end-users perceived that the videoconference provided knowledge or support that could be applied in their clinical practice.

These concepts are closely aligned with the aim of the study and were operationalised through the workshop (phase 1) and the interview guide (phase 2).

#### Phase 1

The 2-h workshop included a presentation of the most frequent patient characteristics (personas) and the typical MRSA pathway (user journey). In the workshop, seven end-users participated, including one patient, three GPs, two IPs and one infectious disease specialist. The first author was the facilitator of the workshop. To address relevance, acceptability and usefulness of end-users, needs and ideas were written on Post-it notes using the think, pair and share method. At the end of the workshop, Post-it notes were grouped into themes by the participants. After the workshop, the first author summarised the data from the workshop and constructed written instructions for the videoconference, which were reviewed by the workshop participants. Comments from the review were taken into consideration and the final content and workflow were accepted by all participants.

The first author composed guidelines and a checklist as supplementary materials to facilitate the videoconference, drawing on the newly developed instructions, practical aspects of telehealth [17] and the Danish MRSA guideline [1].

#### Phase 2

Based on the results of Phase 1, we conducted the specialist-assisted videoconference for MRSA pathways, where the patients, sometimes together with family members and the practice staff (a nurse or a GP) were placed in the GP’s consultation room. The first author (novice in qualitative research) performed all the videoconferences as ‘the specialist’ and made most of the analysis, except for the theoretical understanding of the participants’ statements and their interrelationship. The specialist (first author) was visibly present *via* the practice staff’s computer screen. A total of 11 patients were initially included in the study; however, three patients dropped out following their inclusion. Consequently, eight videoconferences were completed (Table 1). Afterwards, eight patients and seven practice staff (three nurses and four GPs) were interviewed. One interview with a practitioner was cancelled due to sick leave. Patient interviews lasted 15–25 min, while those with practice staff lasted 8–19 min. Between 2 and 7 days after the videoconference, semi-structured telephone interviews were conducted with the participants. To ensure impartiality and foster honest communication, the interviews were conducted by an IP from the Department of Clinical Microbiology at Lillebaelt Hospital, who was not otherwise involved in the study project. The first author developed two versions of semi-structured interview guides, one for patients and one for practice staff, a transcription guide and an analysis guide. In both interviews, the participants were asked about the usefulness of the intervention, the need for access to specialist resources to address relevance, and barriers to and incentives for the use of this specific type of videoconference to address acceptability. For details, see Supplementary File 1. Analysis of the qualitative interviews was conducted by the first author (novice) using NVivo 12 (QSR International, 2014). Interviews were recorded and subsequently transcribed. To structure and reduce the transcribed text, lines were coded. For further text reduction, the codes were merged into themes and subthemes. Next, the matrix in NVivo was used to summarise and condense the data. To analyse the interviews, we used Kvale and Brinkmann’s method [18], which includes three interpretative contexts: The analysis process initially focused on what the participants seemed to perceive as the meaning of their statements (self-understanding), leading to a common sense understanding. In the final step, the theoretical understanding of the statements and their interrelationships was examined to achieve a more comprehensive understanding. This final step was conducted in the discussion section, drawing on relevant research literature and relevant parts of Jody Gittell’s theory of relational coordination [19]. Relational coordination is a process of coordinating work between professionals, which is an essential part when working cross-sectionally. The theory encompasses four communication dimensions (frequent, timely, accurate, problem-solving) and three relational dimensions (shared knowledge, shared goals, mutual respect). Fostering high levels of relational coordination across organisations, in particular, clinical pathways (protocols or guidelines to integrate work), boundary spanners (case managers), patient rounds (meetings), and shared information systems (administrative and clinical) are essential. These components are expected to be more effective when used in conjunction with one another [19]. During the analysis, the communication dimension ‘accuracy’ was used to examine interrelationships and to achieve a more comprehensive understanding. Moreover, the theory was applied to analyse the structure of the videoconference, drawing specifically on the theory’s about the use of guidelines for integrating work.

**Table 1.** Patient characteristics.

Patient^1^	Age group	Gender	Initial infection	Livestock-associated	Family size	Small children in family ^2^	Eczema	Pregnancy	Healthcare worker
1	40–49	Female	Yes	–	4+	–	–	–	–
2	30–39	Female	–	–	3	Yes	–	–	–
3	30–39	Male	Yes	–	2	–	Yes	–	–
4	40–49	Male	Yes	Yes	4+	–	–	–	–
5	30–39	Female	Yes		2	–	–	Yes	–
6	50–59	Female	Yes	–	3	–	–	–	–
7	30–39	Female	–	–	4+	Yes	Yes	–	Yes
8	30–39	Female	–	Yes	3	–	–	–	–

^1^ Three patients from the catchment area of SLB: Lillebaelt Hospital and five from the catchment area of, Hospital of Southern Jutland.

^2^Below 2 years of age.

The quotations were translated into English by a translation bureau. Participant names were changed to unique patient, nurse and GP numbers.

### Approvals and participant consent

Approvals and participant consent were required for Phase 2 only. Data storage and access to patient records were approved by the Region of Southern Denmark (20/25135) and the Danish Patient Safety Authority (S-31-1521-375), respectively. Ethical approval was not necessary according to the Regional Committees on Health Research Ethics for Southern Denmark (S-20192000-155). The patients received oral and written project information, along with a link to sign the informed consent form using NemID (a public secure electronic user login developed by the national authorities in Denmark). Written project information was sent by email to general practices. The practice staff gave oral consent at the beginning of the recorded interview.

## Results

### Phase 1

**Figure 2.**
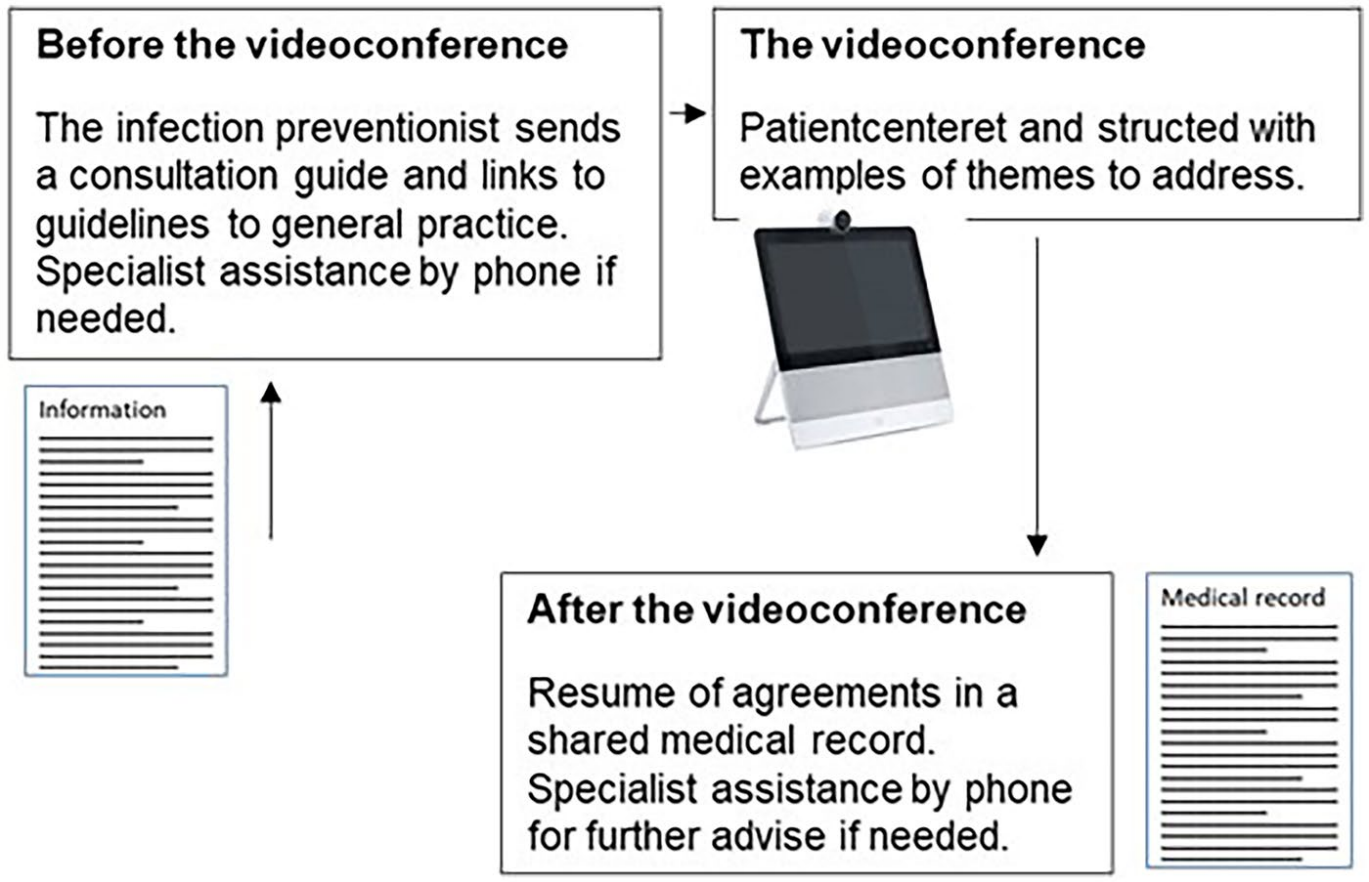
The written description of the videoconference between a remote-sitting specialist, a patient, and a practice staff. *Note:* Further information on the written description is in Supplementary File 2.

The written description of the videoconference consisted of a list of potential themes to be discussed and tasks to be completed before and after the consultation for either the IP or the practice staff. The timeframe was a double consultation (2 × 15 min) according to the organisational structure in general practice clinics. The videoconference was planned shortly after the diagnosis. Unsolved needs were addressed by phone after the videoconference. Questions from patients and tailored pathways were a high priority. For details, see Figure 2 and Supplementary File 2.

### Phase 2

Patient characteristics are displayed in Table 1. The analysis revealed three main themes: (1) ways to collaborate in standard care and videoconferences, (2) shared knowledge and planning of the pathway and (3) barriers when using videoconferences.

### Ways to collaborate in standard care and videoconference

Practice staff found that they handled MRSA care in videoconferences markedly differently from their usual practice. One GP expressed it this way:

*‘We’re a little quick to say drive down to the pharmacy. They have these packages and they tell you everything you need to know’* (GP1). Furthermore, the traditional way using guidelines was stated as follows: ‘*I have used the material that I have been able to find’* (Nurse2). The majority of practice staff obtained specialist advice by phone: *‘There are some questions that I have to say I need to follow up on’ (GP2).* However, when the structure was changed, and an IP actively participated in a real-time dialogue with the patients and the practice staff, it was described as acceptable overall. A GP phrased this as follows: *‘The consultation worked incredibly well. The patient felt really well taken care of. And the fact that the three of us could talk. There was a nice atmosphere, if you can say that about a consultation. It was a good environment to do it in, I think’* (GP3). The participants found their interactions during the videoconference to be respectful of one another’s competencies. Professional identities, specialised knowledge and status differentials did not negatively influence the videoconference. A patient expressed it this way: *‘I actually thought it worked well because they were good at supporting each other. My own nurse knows what she needs to know as part of her job, and the external person, if you can call her that, knows her speciality to the max’* (Patient4), and a GP said: *‘I don’t really think that it is interfering with what we do. I don’t see it that way. I feel it is a help’* (GP1). The complexity of MRSA care was described in several ways by the practice staff. Most reported that patients and their families asked many questions about MRSA, reflecting the complexity of the pathway. A nurse expressed it this way: ‘*Who should be treated and how many members of the family need a swab and who doesn’t need a swab*’ (Nurse2).

### Shared knowledge and planning of the pathway

The participants considered the videoconference thorough and relevant, focusing on patients’ needs and being forward-looking. A patient stated: *‘When the video call was finished, the agreement was made directly with the doctor about when we would come back for the test. So when you left, you had a plan for everything’* (Patient8). A GP explained: *‘Patients got answers to their questions and help with the things they felt were a problem in this situation’* (GP2). Most often, information was passed from the specialist to the practice staff and the patient. Questions addressed included employment conditions in relation to MRSA status, contagiousness, and strategies for MRSA decolonisation. The practice staff played an important role in contributing information about each patient’s medical history and household context. Most patients and practice staff considered the session to be educational. They perceived guidelines for MRSA care as unclear, which led to uncertainty among the practice staff regarding their professional competencies. A nurse expresses it in this way: ‘*Who should be treated and how many members of the family need a swab, and who doesn’t need a swab? That’s where it gets a little tricky for me. Those who work on a pig farm and those who are going to surgery. I think there are many variations anyway’* (Nurse2). A nurse described the educational aspect as follows: ‘*The infection preventionist was good at talking very understandably about what MRSA is and how treatment would proceed if that’s what we chose to do’* (Nurse2). In a few cases, the new information provided during the videoconference led to a change in the practice staff’s approach. The interviews indicated that both written communication in the patient record after the videoconference and the management of future questions by phone were useful and relevant: *‘There was a clear description of what we had gone through. Nothing was missing. I was also given a number to call if I had any questions, and patients were given a number to call. I think it was rounded off well’* (GP2).

### Barriers when using videoconferences

Both patients and practice staff requested a clearer written agenda and a more explicit definition of roles and responsibilities before the videoconference. A GP explained: *‘I was a bit unsure at first whether it was me who was going to run the consultation or whether it was the infection preventionist and what we were going to talk about’* (GP2). In addition, the patients were concerned about receiving written information on MRSA before the meeting. A patient stated: ***‘****I would have liked to have had the material before the consultation so I could have read about MRSA. I feel best when I am prepared for a meeting, because then I can ask my questions properly’* (Patient6). Most patients valued access to specialist healthcare that would otherwise be unavailable. However, the impersonal nature of communication through an online technology was a concern among some patients. A patient expressed the impersonal nature of communication through an online technology as follows: ‘*Video conferencing is not quite the same as sitting in front of someone. It’s easier to talk when you’re sitting next to each other’* (Patient1). Furthermore, in the case of families with many family members, it was difficult for everyone to be visible on the screen. In addition, stationary computer screens made it difficult for the patient to show, for example, eczema to the IP *via* video. However, most caregivers were positive about video communication, even though they had no prior experience with videoconferencing in their practice: *‘It’s also about making the most of the possibilities that exist today – that you can have these video meetings. I haven’t done that before, but we shouldn’t be so afraid. There may be others who work this way, I just haven’t done it before, but it was a boost to my work’* (Nurse 2). Some of the practice staff mentioned that the videoconferences were time-consuming: ‘*If there are any drawbacks, it’s probably that it takes longer. You certainly need to be prepared for that’* (GP2). However, most patients found the intervention time-efficient due to decreased travel time. They also benefitted from the improved access to specialist care: *‘I definitely think this is the best option because it’s nearby, so I don’t have to spend a whole lot of travel time on it. At the same time, I’m sure you get the correct information because you’re talking to a specialist in the field’* (Patient2).

## Discussion

In this study, we developed an intervention involving a face-to-face meeting between the patient and practice staff, with a video connection to a hospital specialist. Overall, the videoconference was considered useful, acceptable and relevant if it was structured, patient-centred and facilitated pathway planning. The experiences of patients and practice staff differed in terms of time spent and their perceptions of communication quality. Some practice staff found the videoconference time-consuming. In contrast, patients found the videoconference efficient. Practice staff perceived the communication quality as high, but some patients preferred face-to-face communication with the hospital specialist.

### Analysis and comparison with existing literature

Relational coordination is a process of coordinating work among professionals, encompassing four communication dimensions – frequency, timeliness, accuracy and problem-solving – and three relational dimensions: shared knowledge, shared goals and mutual respect [19]. According to Gittell, meetings provide a convenient forum for high-quality communication among participants; however, the communication dimension of accuracy may differ in online meetings. Comparing the content and quality of video, telephone and face-to-face consultations in primary care, Hammersley and colleagues found that video consultations may only be suitable for simple problems, whereas face-to-face consultations were the most information-rich [20]. Mascia et al. explored face-to-face communication versus electronic-based tools due to the tendency of clinicians to increasingly rely on emails, text messages and WhatsApp during multidisciplinary team meetings. They concluded that such tools may hinder the quality of group discussions and debates, as senior colleagues frequently use these messaging apps, potentially leading to reduced attention and distractions [21]. This supports the experiences of some patients in this study, who preferred face-to-face communication with hospital specialists. In our intervention, however, we mitigated the limitations of two-way videoconferences, as the patient and practice staff had a face-to-face consultation while the specialist joined *via* a video link. This approach helped to avoid issues such as limited access for patients unable to use online technologies [11,22]. A newly published survey asked patients about their preferred type of outpatient appointment. The study found that most patients preferred a videoconference with a specialist doctor in a local GP clinic or small hospital over an in-person consultation with a GP [23]. However, currently, a videoconference is only available in general practice clinics between GPs and patients through the application ‘My Doctor’ in Denmark [24] since a common infrastructure for peer-to-peer cross-sector videoconferences has yet to be developed.

According to Gittell, a written description of clinical pathways helps capture lessons learned from previous experiences, allowing a process to be replicated without ‘reinventing the wheel’ [20]. The videoconference was structured and included potential themes to be addressed and tasks to be completed before and after the videoconference. Results from previous research examining factors that contribute to successful cross-sector partnerships indicate that vague structures and unclear roles harm productivity [25], which supports the structured approach used in our videoconference model. Previous research has not examined this standardised approach to managing consultations in general practice. However, given the anticipated increase in patient pathway complexity and the transfer of activities from secondary to primary care, this finding is significant. However, our results require confirmation in further studies.

Our study differs from most existing studies in the field of telemedicine in general practice, as the majority of published studies focus on videoconsultations where the patient is remote from the clinician. However, we identified two studies on videoconferencing in general practice involving hospital specialists. Our results are largely in line with the previously mentioned Danish study involving cancer patients and GPs [5] and a study from the United Kingdom [26]. The latter examined the interaction between patients, practice staff and a specialist during diagnosis and decision-making in a videoconference. Cross-boundary collaboration enabled practice staff to develop their skills and actively participate in diagnosis and decision-making. However, interprofessional interaction has been observed to limit patient involvement [20]. Our intervention focused on patient needs. To minimise interprofessional communication during the videoconference, practice staff were informed of MRSA guidelines beforehand. Additionally, they could contact the IP by phone before and after the videoconference, which may have encouraged more patient-centred meetings.

### Strengths and limitations

A key strength of this study was user involvement, which is central to the participatory design method. Users were engaged as active partners both in planning the videoconference and in its real-world implementation. However, involving only one patient in the planning process (phase 1) may limit the ability to fully capture patient needs. Another strength was that an independent IP and not the principal investigator conducted the interviews, allowing participants to speak freely during the interview. However, certain limitations must be considered. First, participant recruitment was challenging due to strict inclusion criteria and the COVID-19 pandemic at that time, which led to fewer visits to general practice clinics and a decline in MRSA cases. Consequently, the study involved only small-scale testing, with 17 interviews and eight videoconferences. This raises concerns about the transferability of findings and the extent to which they capture the needs of end-users. Second, a major concern is the fact that the first author served as the principal investigator, facilitated the workshop, acted as the specialist during the videoconferences, and conducted the first steps of the analysis at a novice level. This likely introduced bias, as the first author’s prior understanding of the benefits of integrating specialist competencies into general practice *via* videoconferencing may have led to an overestimation of its usefulness, relevance and acceptability. Additionally, incorporating reflections in the first steps of the analysis from other senior researchers could have strengthened the rigour and robustness of the findings, as the first author made most of the analysis, except for the theoretical understanding of the participants’ statements and their interrelationship. Finally, a note of caution about analysing the interviews of patients and practice staff jointly.

### Perspectives

According Clemensen and colleagues the participatory design methodology includes: needs assessment, idea generation, testing and retesting, and evaluation [13]. The evaluation, which is not carried out in the present study, should, according to Clemensen and colleagues, include an assessment of the effectiveness of care examined in clinical trials and cost-effectiveness studies. Further research is still necessary as preparation for a randomised controlled trial.

Most importantly, relevant patient outcomes should be tested and assessed in a pilot trial comparing outcomes such as infection rates, rates of successful decolonisation treatment (MRSA-free) and stigma rates between standard care and the videoconference developed in this study. The intervention is relatively resource-intensive, both due to the videoconference’s timespan and the fact that several health personnel have to use their time together. Therefore, the pilot trial must furthermore focus on the time-consuming part due to limited resources in the healthcare system. Depending on the results from the pilot trial, a large-scale randomised controlled trial can be planned and conducted.

Our findings on enhancing access to specialist care in the primary healthcare sector through videoconferencing may also be beneficial for managing other low-prevalence diseases or complex healthcare pathways.

## Conclusion

Videoconferences are feasible and were perceived as useful, acceptable and relevant when they were structured, patient-centred and facilitated pathway planning. However, some patients found them less information-rich compared to face-to-face meetings, and some practice staff considered them time-consuming.

## Supplementary Material

Supplementary File 1.docx

Supplementary File 2.docx
