# Supplementary material for: Videoconferences between remote-sitting specialist, patient and practice staff concerning low prevalent diseases and complex pathways in general practice clinics: a feasibility study
Source: Scand J Prim Health Care. 2026 May 5;44(1):2666626. doi: 10.1080/02813432.2026.2666626 (PMC13148079; doi:10.1080/02813432.2026.2666626)
Supplement: Supplementary File 1.docx [file IPRI_A_2666626_SM2275.docx]

Supplementary File 1

**Interview guide for telephone interviews with patients**

**Introduction:**

- Thank you for participating
- The interviewer introduces themselves briefly
- Brief overview of the interview framework
- Duration approximately 15-20 minutes
- Anonymous
- The conversation will be recorded
- Do you have any questions before we begin?

**Is the intervention useful and accepted from the patient's perspective?**

- What has been important for you regarding MRSA and help from your general practice clinics?
- Have you experienced that your needs were addressed? How? Can you give some examples?
- How did you feel about the consultation taking place in your general practice clinics and not at the hospital?
- How did you find the booking for the videoconference (the consultation where the infection preventionist was present via video)?
- How did you experience the videoconference itself? Can you remember what was discussed? Was there anything missing?
- How did you perceive the time frame? Was there any wasted time (including waiting time)?
- Would you use the opportunity for a phone call with an infection preventionist after the shared videoconference? Why/why not?

**Is it useful and accepted to use specialist resources in general practice clinics via video connection from the patient's perspective?**

- How did you perceive having an extra person infection preventionist via video?
- How did the infection preventionist contribute? Did it add anything extra that you needed? Why/why not? Can you give some examples?

**What barriers and incentives are there for the intervention from the patient's perspective?**

- How do you think this form of cooperation between the staff at the general practice clinic and the infection preventionist at the hospital works?
- What disadvantages do you think there are?
- Would it deter you from accepting the offer another time?
- Did you experience any clear advantages of the hospital and general practice clinics collaborating in this way?
- If you wish to accept the offer another time, what would be the reason?
- Was there anything you experienced as unpleasant? How?
- Do you have suggestions on how help from your general practice clinic and hospital could be improved?

**Conclusion of the Interview:**

- Do you have questions or comments on any of the topics we discussed?
- Is there anything I haven't asked that you think is important to include?

**Interview guide for telephone interviews with practice staff**

**Introduction:**

- Thank the interviewee for participating.
- Briefly introduce the interviewer.
- Outline the framework of the interview.
- Duration: approximately 10 minutes.
- Ensure anonymity.
- The conversation will be recorded.
- Consent via audio recording: Ask for the name of the practice staff and if they agree to the interview being used for the research project (this must be included in the audio recording as documentation).
- Do you have any questions before we begin?

**Is the intervention useful and accepted from the perspective of the general practice clinics?**

- Overall, what do you think of the concept?
- How do you feel about the content of the videoconference? Did we discuss the most important things? What was most important?
- Was the time appropriate? (Waste time/waiting time)
- How has the written communication worked (e.g., sharing video links and correspondence notes)?
- Do you expect to need phone advice after the shared videoconference? If yes – which areas do you expect to need advice on?

**Is it useful and accepted to use specialist resources in general practice clinics via video connection from the perspective of the general practice clinics?**

- What do you think of the cooperation and the tasks you had?
- Is there a need for collaboration around patients with MRSA? If so, how much sparring is needed?
- How did you feel about having a specialist involved in the consultation with the patient? Are there clear benefits or disadvantages?
- How did you perceive the quality of expertise from the nurse?
- What do you think about the correspondence notes from the infection preventionist? (The fact that they are sent and how quickly they are available)

**What barriers and incentives are there for the intervention from the perspective of the general practice clinic?**

- Would you choose to use the concept if offered another time? Why?
- Can you think of anything that would make you choose not to use it (e.g., telephone and written advice is sufficient, economic or technical issues)?
- Do you think the basis for offering the concept is in place, or do you have suggestions for changes?

**Conclusion of the Interview:**

- Do you have questions or comments about any of the topics we discussed?
- Is there anything I have not asked that you believe is important to include?
