# Supplementary material for: Videoconferences between remote-sitting specialist, patient and practice staff concerning low prevalent diseases and complex pathways in general practice clinics: a feasibility study
Source: Scand J Prim Health Care. 2026 May 5;44(1):2666626. doi: 10.1080/02813432.2026.2666626 (PMC13148079; doi:10.1080/02813432.2026.2666626)
Supplement: Supplementary File 2.docx [file IPRI_A_2666626_SM2274.docx]

Supplementary File 2

| Table 1. Actions before the videoconference |
| --- |
| - The practice staff invites and informs the patient about the videoconference by phone. - The infection preventionist sends a consultation guide to the practice staff, including a list of potential themes to address during the videoconference, tasks to be accomplished before and after the videoconference, and links to MRSA guidelines. - To avoid interprofessional talk during the videoconference, the practice staff can call the infection preventionist for advice. - The infection preventionist sends a consultation guide to the patient, which includes a list of potential themes to address during the videoconference. |

| Table 2. List of potential themes to address during the videoconference |
| --- |
| - Introduction and purpose of the videoconference. - A summary of the patient's MRSA care program. - Questions (patient and family) and delivery of written information in print or electronically. - Information matching patients' needs (e.g., livestock-associated MRSA, MRSA in newborns and children up to two years of age, healthcare workers, pregnancy, individual risk factors such as e.g. eczema or wounds, frequent hospitalization, psychosocial issues). - Planning of the MRSA care program (treatment of any clinical infections, clarifying carrier status, treatment of, for example, wound/eczema, decolonization treatment, MRSA follow-up swabs one and six months after treatment, closing the MRSA care program). - Considering the need for further support due to life circumstances or complexity (involvement of municipalities e.g., social nurse, telephone conversation with specialist expertise in MRSA, consultation with the practice staff). - Summarizing the output of the videoconference to facilitate a common understanding of agreements. |

| Table 3. Actions after the videoconference |
| --- |
| - The infection preventionist writes a summary in the hospital's electronic patient record, with a copy sent electronically to the general practice. - The infection preventionist is available for advice by telephone for patients and practice staff. - The practice staff ensures that consultations for MRSA follow-up swabs are booked, writes prescriptions for MRSA decolonization treatment for all family members, and reacts after treatment in case of treatment failure, and closes the MRSA care program when the family is effectively treated (considered MRSA-free). |
